# Diagnostic Accuracy of Ultrasonography in the Detection of Subacromial Bursitis Using Magnetic Resonance Imaging as the Gold Standard

**DOI:** 10.7759/cureus.112955

**Published:** 2026-07-19

**Authors:** Muqaddas Sana, Jawairia Arif, Muhammad Umair, Tooba Anjum, Sadia Anwar, Irum Shahzad, Muhammad Hamid Chaudhary

**Affiliations:** 1 Diagnostic Radiology, Mayo Hospital, Lahore, PAK; 2 Radiology, THQ Hospital Safdarabad, Sheikhupura, PAK; 3 Radiology, INMOL Cancer Hospital, Lahore, PAK; 4 Radiology, Mayo Hospital, Lahore, PAK; 5 Cardiac Surgery, Chaudhry Pervaiz Elahi Institute of Cardiology, Multan, PAK

**Keywords:** diagnostic accuracy, magnetic resonance imaging, rotator cuff pathology, shoulder pain, subacromial bursitis, ultrasonography

## Abstract

Background: Subacromial bursitis is a frequent cause of shoulder pain and functional disability and is commonly associated with rotator cuff pathology. Accurate imaging is essential for diagnosis. Ultrasonography is widely used for its accessibility and cost-effectiveness compared with magnetic resonance imaging (MRI), which is considered the gold standard for detailed soft tissue assessment.

Aim: This study aimed to determine the diagnostic accuracy of ultrasonography in detecting subacromial bursitis using MRI as the reference standard and to assess its association with demographic and clinical factors.

Methods: This cross-sectional diagnostic accuracy study was conducted in the Department of Diagnostic Radiology, Mayo Hospital, Lahore, Pakistan, from January 2025 to January 2026. A total of 134 patients aged 20-60 years with clinically suspected subacromial bursitis were included through non-probability consecutive sampling. All patients underwent ultrasonographic examination followed by MRI of the shoulder on the same day. The sample size was calculated based on an anticipated specificity of 77.8%, an expected disease prevalence of 70%, a 95% confidence level, and a 13% absolute precision. Diagnostic performance parameters, including sensitivity, specificity, positive predictive value (PPV), negative predictive value (NPV), and overall diagnostic accuracy, were calculated using MRI as the reference standard. Statistical analysis also included odds ratio, stratification, normality testing, and chi-squared test for association.

Results: Ultrasonography demonstrated a sensitivity of 76.3%, a specificity of 82.9%, a PPV of 91%, an NPV of 60.7%, and an overall diagnostic accuracy of 78.4% in detecting subacromial bursitis. A strong association was observed between ultrasound and MRI findings for subacromial bursitis (OR=15.68; 95% CI: 6.10-40.28; p<0.001), with moderate agreement between both modalities (Cohen's kappa=0.54). The prevalence and severity of subacromial bursitis and associated rotator cuff pathologies increased with age. Stratified analysis showed higher ultrasound sensitivity in younger patients (≤40 years), whereas specificity was higher in older patients (>40 years).

Conclusion: Ultrasonography demonstrated good diagnostic accuracy in detecting subacromial bursitis when compared with MRI. Its high PPV and moderate agreement with MRI findings support its use as an initial imaging modality for the evaluation of suspected subacromial bursitis. An increase in subacromial and associated rotator cuff abnormalities was observed with age. Ultrasonography provides a practical, accessible, and cost-effective imaging option, particularly in resource-limited settings. However, MRI should be considered in equivocal cases or when clinical suspicion persists despite a negative ultrasound examination.

## Introduction

Subacromial bursitis is an important cause of shoulder pain, is often linked to rotator cuff pathology and impingement syndrome, and adds significantly to musculoskeletal morbidity across the globe [1]. Epidemiological studies have shown that almost 7-26% of the general population experience shoulder pain at any given time, with about 44-65% of these cases being subacromial disorders [2]. The subacromial bursa is an important biomechanical structure that facilitates smooth glenohumeral and subacromial movement, and inflammation causes pain, limitation, and functional deficit [3]. Recurrent overhead tasks, degenerative alterations, and rotator cuff tendinopathy are usually associated with chronic bursal inflammation. Subacromial bursitis is difficult to diagnose clinically because its symptoms overlap with those of other shoulder diseases, such as rotator cuff tear and adhesive capsulitis [4]. Subacromial bursitis is highly prevalent and affects function, and consequently, a precise and prompt diagnosis is needed to optimize treatment efficacy and minimize disability. As a result, imaging modalities have become important diagnostic adjuncts and inform management approaches [5].

Magnetic resonance imaging (MRI) is widely regarded as the reference standard for evaluating shoulder soft tissue pathology, such as subacromial bursitis, because it offers high contrast resolution and multiplanar imaging [6]. Bursal fluid and synovial thickening, as well as related rotator cuff pathophysiology, can be viewed in great detail with the help of MRI, which allows performing a thorough examination. Previous studies have reported that MRI is 90% sensitive in identifying subacromial bursitis especially when there is an associated rotator cuff pathology [7]. Nevertheless, there are associated limitations, such as high cost, inaccessibility, and longer acquisition times, which could hinder its frequent use in resource-constrained facilities [8]. There are also contraindications (presence of metallic implants and claustrophobia in patients) that further limit its applicability. Although MRI has certain diagnostic superiority, its use in all suspected cases is neither viable nor cost-effective in most healthcare sectors [9].

The use of ultrasound in musculoskeletal disorders has proven successful due to its real-time imaging capability, portability, and affordability [10]. Ultrasound images in subacromial bursitis can identify bursal thickening, fluid buildup, and dynamic impingement of the shoulder during movement. Past research has reported sensitivities of 79-96% and specificities of 85-98% for ultrasound in detecting subacromial bursitis compared with MRI [11]. The flow characteristics of ultrasound enable the evaluation of functional anomalies not seen on static imaging. Moreover, interventions performed using ultrasound, including ultrasound-guided bursal injections, are more diagnostic and more effective for treatment [12]. Dependence of the operator is still a significant weakness whereby diagnostic reliability entirely depends on the skill of the sonographer. In spite of this, the development of high-frequency transducers has significantly enhanced image resolution and diagnostic reliability [13].

In Pakistan, musculoskeletal disorders have a heavy burden because of occupational risk factors, inadequate ergonomic knowledge, and late healthcare-seeking behavior [14]. Financial and infrastructural constraints also restrict access to high-level imaging modalities such as MRI in most public sector hospitals [15]. Ultrasound, on the other hand, is very common, inexpensive, and commonly used in regular clinical practice in tertiary care facilities [16]. Although it is common in bypass, there is limited local evidence comparing the diagnostic accuracy of ultrasound on subacromial bursitis with MRI as a gold standard. Variations in disease presentation, healthcare facilities, and provider expertise highlight the need for population-specific studies to validate findings reported in the global literature. Moreover, determining the validity of ultrasound would be a major step towards minimizing the use of MRI, thereby reducing healthcare expenses and enhancing patient accessibility. This evidence is essential for streamlining diagnostic routes and resource utilization in the Pakistani healthcare system. Thus, the objective of the study is to assess the accuracy of ultrasound for diagnosing subacromial bursitis, using MRI as the reference standard, in a Pakistani population.

## Materials and methods

Study design and setting

This cross-sectional diagnostic accuracy study was conducted in the Department of Diagnostic Radiology, Mayo Hospital, Lahore, Pakistan, from January 2025 to January 2026.

Sample population

To obtain a sample size of 134 patients, the level of confidence was taken to be 95%, the margin of error 13%, the anticipated diagnostic accuracy of ultrasonography 80.4%, the sensitivity of ultrasonography 81.6%, the specificity 77.8%, and the prevailing prevalence of subacromial bursitis 70%.

To obtain a sample size of 134 patients, the level of confidence was taken to be 95% with the margin of error being 13%. Due to the anticipated diagnostic accuracy of ultrasonography being 80.4%, the sensitivity of ultrasonography is reported to be 81.6%, the specificity 77.8%, and the prevailing prevalence of subacromial bursitis 70%. Patient sampling used the non-probability consecutive sampling technique. The study included patients of either gender, aged 20-60 years, who were clinically suspected of having subacromial bursitis according to the predefined operational criteria.** **The study exclusion criteria included patients who had surgery on the same shoulder previously, shoulder dislocation, shoulder girdle fracture, neoplastic lesions, or congenital anomalies.

Data collection

A total of 134 patients fulfilling the inclusion criteria underwent ultrasonographic examination of the affected shoulder using a high-frequency linear-array transducer (7-12 MHz). Patients were examined in the seated position. The subacromial-subdeltoid bursa and rotator cuff tendons were evaluated in longitudinal and transverse scanning planes. Dynamic assessment was performed during active shoulder abduction. Subacromial-subdeltoid bursal fluid was measured perpendicular to the bursal margins at the point of maximum distension. Ultrasonographic diagnosis of subacromial bursitis was based on bursal fluid distension >2 mm, with or without bursal wall thickening. Dynamic impingement was defined as abnormal bunching of the subacromial-subdeltoid bursa or failure of smooth passage of the supraspinatus tendon and bursa beneath the acromion during active shoulder abduction [9,17]. Color Doppler was not routinely used to diagnose subacromial bursitis.

All patients underwent MRI of the affected shoulder on the same day as the ultrasonographic examination. MRI was performed on a 1.5-T scanner using a dedicated shoulder coil. Images were obtained in axial, oblique coronal, and oblique sagittal planes. The imaging protocol included T1- and T2-weighted fat-suppressed and proton-density fat-suppressed sequences, with a slice thickness of 3-4 mm. Intravenous contrast was not administered. Subacromial-subdeltoid bursitis on MRI was defined as abnormal bursal fluid distension measuring >2 mm at the point of maximum perpendicular thickness. Bursal fluid demonstrated low signal intensity on T1-weighted images and high signal intensity on fluid-sensitive sequences. Associated bursal wall or synovial thickening and rotator cuff abnormalities were also documented [17,18].

Ultrasonography and MRI were interpreted independently by the same senior radiologist. MRI findings were assessed without reference to the ultrasonographic findings. All imaging findings were recorded by the principal investigator on a predesigned data collection form.

Data analysis

All the obtained data were tabulated and analyzed in IBM SPSS Statistics for Windows, Version 27.0 (IBM Corp., Armonk, New York, United States). Quantitative variables like age were reported as the mean±SD, whereas categorical variables like gender and the presence of a subacromial bursitis as noted through ultrasonography and MRI were reported as frequencies and percentages, respectively. Sensitivity, specificity, positive predictive value (PPV), negative predictive value (NPV), and diagnostic accuracy were also determined using typical formulas and provided as illustrated below. Diagnostic accuracy was further stratified by age, gender, body mass index (BMI), and the affected shoulder side to assess the potential influence of these variables. After the stratification, the chi-squared tests were used, and a p-value of 0.05 or less was taken as statistically significant.

Ethical considerations

The Institutional Review Board of King Edward Medical University (approval number: 181/RC/KEMU) granted ethical approval for the study before its commencement. All participants signed informed consent forms after they had been informed about the purpose of the study, the procedures of the study, the risks involved, and the benefits of the study. Patient confidentiality was maintained throughout the study, and all procedures were conducted in accordance with the principles of the Declaration of Helsinki.

## Results

A total of 134 patients were included in the study. The largest age groups were 41-50 years and 51-60 years, each comprising 38 patients (28.4%). There were 77 male patients (57.5%) and 57 female patients (42.5%). The right shoulder was involved in 73 patients (54.5%) and the left shoulder in 61 (45.5%). The most common BMI category was <25 kg/m², seen in 57 patients (42.5%) (Table 1).

**Table 1:** Demographic and clinical characteristics of the study population (n=134)

Variable	Category	Frequency (n)	Percentage (%)
Age group (years)	20-30	27	20.1
	31-40	31	23.1
	41-50	38	28.4
	51-60	38	28.4
Gender	Male	77	57.5
	Female	57	42.5
Body mass index (kg/m²)	<25	57	42.5
	25-30	37	27.6
	>30	40	29.9
Side involved	Right	73	54.5
	Left	61	45.5

The mean age of the study population was 42.6±11.1 years, while the mean BMI was 26.8±4.6 kg/m². Shapiro-Wilk tests indicated normal distributions for age (p=0.071) and BMI (p=0.065), allowing the use of parametric statistical tests (Table 2).

**Table 2:** Normality assessment of continuous variables

Variable	Mean±SD	Shapiro-Wilk p-value	Distribution
Age	42.6±11.1	0.071	Normal
Body mass index	26.8±4.6	0.065	Normal

On ultrasonography, subacromial bursitis was detected in 78 patients. Its frequency increased from 11 patients (40.7%) in the 20-30-year age group to 23 patients (60.5%) in the 51-60-year age group. Bursal bunching, supraspinatus tendinosis, and long head of biceps pathology were also more frequent in older age groups (Table 3).

**Table 3:** Ultrasound findings stratified by age groups Ultrasound-detected tendon tears were recorded as present or absent. Tear thickness and grade were not classified into partial- or full-thickness categories in the original data collection; therefore, the reported tear findings represent any sonographically detected tendon tear.

Pathology	20-30 years (n=27)	31-40 years (n=31)	41-50 years (n=38)	51-60 years (n=38)
Subacromial bursitis	11 (40.7)	20 (64.5)	24 (63.2)	23 (60.5)
Bursal bunching	17 (63)	20 (64.5)	31 (81.6)	33 (86.8)
Supraspinatus tear (any tear)	17 (63)	22 (71)	26 (68.4)	28 (73.7)
Infraspinatus tear	8 (29.6)	13 (41.9)	19 (50)	25 (65.8)
Subscapularis tear	8 (29.6)	15 (48.4)	23 (60.5)	25 (65.8)
Supraspinatus tendinosis	15 (55.6)	18 (58.1)	29 (76.3)	33 (86.8)
Infraspinatus tendinosis	13 (48.1)	16 (51.6)	19 (50)	29 (76.3)
Subscapularis tendinosis	10 (37)	15 (48.4)	20 (52.6)	23 (60.5)
Long head of biceps pathology	14 (51.9)	14 (45.2)	27 (71.1)	35 (92.1)

MRI showed subacromial bursitis in 93 patients. The frequency was higher in patients above 40 years, with 32 cases (84.2%) in the 41-50-year age group and 31 cases (81.6%) in the 51-60-year age group. MRI findings of partial supraspinatus tear, joint effusion, subdeltoid bursitis, acromioclavicular joint arthropathy, biceps tendinopathy, and full-thickness supraspinatus tear also showed an increasing trend with age (Table 4).

**Table 4:** MRI findings stratified by age MRI: magnetic resonance imaging

Parameter	20-30 years	31-40 years	41-50 years	51-60 years
Subacromial bursitis	1.2±0.4	1.5±0.5	1.8±0.6	2.1±0.7
Partial supraspinatus	0.8±0.3	1.2±0.4	1.6±0.5	1.9±0.6
Joint effusion	0.6±0.2	0.9±0.3	1.3±0.4	1.6±0.5
Subdeltoid bursitis	0.7±0.3	1.0±0.4	1.4±0.5	1.7±0.6
Acromioclavicular joint arthropathy	0.5±0.2	0.8±0.3	1.2±0.4	1.6±0.5
Biceps tendinopathy	0.6±0.3	1.1±0.4	1.5±0.5	1.8±0.6
Full-thickness supraspinatus	0.2±0.1	0.4±0.2	0.8±0.3	1.2±0.4

Using MRI as the reference standard, ultrasonography showed 71 true positives, seven false positives, 22 false negatives, and 34 true negatives (Table 5).

**Table 5:** Contingency table of ultrasound versus MRI for the detection of subacromial bursitis MRI: magnetic resonance imaging

Ultrasound finding	MRI positive, n (%)	MRI negative, n (%)	Total, n (%)
Positive	71 (52.9)	7 (5.2)	78 (58.2)
Negative	22 (16.4)	34 (25.4)	56 (41.8)
Total	93 (69.4)	41 (30.6)	134 (100)

The sensitivity of ultrasonography was 76.3% (95% CI: 66.8-83.8), specificity was 82.9% (95% CI: 68.7-91.5), PPV was 91% (95% CI: 82.6-95.6), NPV was 60.7% (95% CI: 47.6-72.4), and overall diagnostic accuracy was 78.4% (95% CI: 70.6-84.5) (Table 6).

**Table 6:** Diagnostic accuracy of ultrasound using MRI as the reference standard

Parameter	Value (%)	95% CI
Sensitivity	76.3	66.8-83.8
Specificity	82.9	68.7-91.5
Positive predictive value	91.0	82.6-95.6
Negative predictive value	60.7	47.6-72.4
Diagnostic accuracy	78.4	70.6-84.5

A statistically significant association was found between ultrasound and MRI findings for subacromial bursitis (OR=15.68; 95% CI: 6.10-40.28; p<0.001). Cohen's kappa value was 0.54, indicating moderate agreement between the two imaging modalities (Table 7).

**Table 7:** Association and agreement between ultrasound and MRI findings MRI: magnetic resonance imaging

Parameter	Value
Odds ratio	15.68
95% CI	6.10-40.28
P-value	<0.001
Cohen's kappa	0.54

In stratified analysis, ultrasonography showed a sensitivity of 83.3% (95% CI: 66.4-92.7) in patients aged ≤40 years and 73% (95% CI: 61.0-82.4) in those aged >40 years. Specificity was 78.6% (95% CI: 60.5-89.8) and 92.3% (95% CI: 66.7-98.6), respectively. Overall diagnostic accuracy did not differ significantly between patients aged ≤40 years and those aged >40 years (81% vs. 76.3%; p=0.534). In contrast, diagnostic accuracy was significantly higher in females than in males (91.2% vs. 68.8%; p=0.003) (Table 8). However, the subgroup estimates had relatively wide confidence intervals and should be interpreted cautiously.

**Table 8:** Stratified diagnostic accuracy of ultrasound by age and gender

Variable	Sensitivity, % (95% CI)	Specificity, % (95% CI)	Positive predictive value, % (95% CI)	Negative predictive value, % (95% CI)	Accuracy, % (95% CI)
Age ≤40 years	83.3 (66.4-92.7)	78.6 (60.5-89.8)	80.6 (63.7-90.8)	81.5 (63.3-91.8)	81.0 (69.1-89.1)
Age >40 years	73.0 (61.0-82.4)	92.3 (66.7-98.6)	97.9 (88.9-99.6)	41.4 (25.5-59.3)	76.3 (65.6-84.5)
Male	64.2 (50.7-75.7)	79.2 (59.5-90.8)	87.2 (73.3-94.4)	50.0 (34.8-65.2)	68.8 (57.8-78.1)
Female	92.5 (80.1-97.4)	88.2 (65.7-96.7)	94.9 (83.1-98.6)	83.3 (60.8-94.2)	91.2 (81.1-96.2)

## Discussion

This study was conducted to determine the diagnostic accuracy of ultrasonography in detecting subacromial bursitis using MRI as the reference standard and to assess its relationship with age-related rotator cuff pathology. The findings showed that ultrasonography had an overall diagnostic accuracy of 78.4%, with a sensitivity of 76.3% and a specificity of 82.9%. The PPV was high (91%), indicating that positive ultrasound findings strongly correlated with MRI-confirmed pathology, whereas the relatively lower NPV (60.7%) suggests that some cases may be missed on ultrasonography. These findings support the utility of ultrasound as a reliable initial diagnostic modality in evaluating subacromial bursitis.

The diagnostic performance observed in this study is comparable to previously published literature. Hassanein et al. reported sensitivity ranging from 79% to 96% and specificity between 85% and 98% for ultrasonography in similar shoulder pathologies [17]. Likewise, Li et al. reported an ultrasound sensitivity of 91% in rotator cuff-related shoulder disorders [18]. The slightly lower sensitivity observed in the present study may be attributed to operator dependency and the difficulty in detecting subtle or early-stage pathology. However, the high PPV indicates strong clinical reliability in cases with positive ultrasound findings.

The present study also demonstrated age-related progression of subacromial and rotator cuff pathology. Ultrasound findings showed increasing frequencies of bursitis, bursal distension, tendinosis, and long head of biceps pathology with advancing age. These findings are in agreement with previous reports showing that degenerative shoulder pathologies increase significantly after the fourth decade of life [19,20]. This age-related progression reflects the cumulative biomechanical stress and degenerative changes affecting the shoulder joint over time.

A significant association was observed between ultrasound and MRI findings for subacromial bursitis, with an odds ratio of 15.68 (95% CI: 6.10-40.28; p<0.001). This indicates a strong relationship between positive ultrasound findings and MRI-confirmed pathology. In addition, Cohen's kappa of 0.54 indicated moderate agreement between the two modalities. These findings are consistent with previous studies showing good concordance between ultrasound and MRI for shoulder pathologies [21,22]. The inclusion of kappa analysis in the present study strengthens the reliability assessment of ultrasound.

The study also highlighted the coexistence of multiple tendon pathologies. Supraspinatus tendinosis was among the most frequent findings, particularly in older age groups, while infraspinatus and subscapularis involvement also showed increasing frequency with age. Long head of biceps pathology was notably high in older individuals. These findings emphasize that subacromial bursitis often exists as part of a broader degenerative shoulder disease process rather than as an isolated pathology [23,24].

Stratified analysis showed variation in diagnostic performance across demographic groups. Interestingly, ultrasound sensitivity was higher in younger patients (≤40 years), whereas specificity was greater in older patients (>40 years). Female patients showed slightly better overall diagnostic accuracy compared to males. These findings suggest that patient characteristics may influence the diagnostic performance of ultrasonography and should be considered during interpretation [25,26].

MRI provided a more detailed assessment of subtle and concurrent shoulder abnormalities because of its superior soft tissue contrast. In this study, MRI was used as the reference standard for comparison with ultrasonography. However, MRI findings were not confirmed by arthroscopy or subsequent clinical follow-up, which should be considered when interpreting the diagnostic performance estimates. Given its accessibility, lower cost, and non-invasive nature, ultrasonography remains a valuable initial imaging modality, particularly in resource-limited settings [27,28].

This study has some limitations. The sample size was adequate for preliminary evaluation but may limit generalizability. Non-probability consecutive sampling may introduce selection bias. All imaging interpretations were performed by a single radiologist, which minimized interobserver variability but limited assessment of reproducibility. Furthermore, ultrasound remains operator-dependent, and its diagnostic performance may vary with experience. Despite these limitations, the study provides useful evidence regarding the clinical utility of ultrasonography in subacromial bursitis.

## Conclusions

Ultrasonography demonstrated good diagnostic accuracy in detecting subacromial bursitis when compared with MRI. The high PPV and significant association with MRI findings support its role as an initial imaging modality. An increase in subacromial and associated rotator cuff abnormalities was observed with age on both ultrasound and MRI. However, the false-negative findings and relatively low NPV indicate that a negative ultrasound examination may not reliably exclude subacromial bursitis. MRI should be considered in equivocal cases or when clinical suspicion persists despite negative ultrasonographic findings. Ultrasonography remains a practical, cost-effective, and accessible imaging modality, particularly in resource-limited settings.
